# *Nontypeable* Haemophilus Influenzae Infection Upregulates the NLRP3 Inflammasome and Leads to Caspase-1-Dependent Secretion of Interleukin-1β — A Possible Pathway of Exacerbations in COPD

**DOI:** 10.1371/journal.pone.0066818

**Published:** 2013-06-26

**Authors:** Johannes Rotta detto Loria, Kristina Rohmann, Daniel Droemann, Peter Kujath, Jan Rupp, Torsten Goldmann, Klaus Dalhoff

**Affiliations:** 1 Medical Clinic III, University Clinic of Schleswig-Holstein, Campus Luebeck, Luebeck, Germany; 2 Institute of Medical Microbiology and Hygiene, University Clinic of Schleswig-Holstein, Campus Luebeck, Luebeck, Germany; 3 Department of Surgery/Thoracic Surgery, University Clinic of Schleswig-Holstein, Campus Luebeck, Luebeck, Germany; 4 Clinical and Experimental Pathology, Research Center Borstel, Borstel, Germany; 5 Airway Research Center North (ARCN), Member of the German Center for Lung Research (DZL), Borstel, Germany; University of California Merced, United States of America

## Abstract

**Rationale:**

*Nontypeable* Haemophilus influenzae (NTHi) is the most common cause for bacterial exacerbations in chronic obstructive pulmonary disease (COPD). Recent investigations suggest the participation of the inflammasome in the pathomechanism of airway inflammation. The inflammasome is a cytosolic protein complex important for early inflammatory responses, by processing Interleukin-1β (IL-1β) to its active form.

**Objectives:**

Since inflammasome activation has been described for a variety of inflammatory diseases, we investigated whether this pathway plays a role in NTHi infection of the airways.

**Methods:**

A murine macrophage cell line (RAW 264.7), human alveolar macrophages and human lung tissue (HLT) were stimulated with viable or non-viable NTHi and/or nigericin, a potassium ionophore. Secreted cytokines were measured with ELISA and participating proteins detected via Western Blot or immunohistochemistry.

**Measurements and Main Results:**

Western Blot analysis of cells and immunohistochemistry of lung tissue detected the inflammasome key components NLRP3 and caspase-1 after stimulation, leading to a significant induction of IL-1β expression (RAW: control at the lower detection limit vs. NTHi 505±111pg/ml, p<0.01). Inhibition of caspase-1 in human lung tissue led to a significant reduction of IL-1β and IL-18 levels (IL-1β: NTHi 24 h 17423±3198pg/ml vs. NTHi+Z-YVAD-FMK 6961±1751pg/ml, p<0.01).

**Conclusion:**

Our data demonstrate the upregulation of the NRLP3-inflammasome during NTHi-induced inflammation in respiratory cells and tissues. Our findings concerning caspase-1 dependent IL-1β release suggest a role for the inflammasome in respiratory tract infections with NTHi which may be relevant for the pathogenesis of bacterial exacerbations in COPD.

## Introduction

NTHi is the most important bacterial pathogen in acute COPD exacerbations (AE-COPD) [1]. Lungs of patients with COPD are often colonized by NTHi, but infections with new strains play an important role in the development of AE-COPD [2]. The activation of the pulmonary immune system by this microorganism may therefore influence the course of acute exacerbations as well as chronic airway inflammation.

In COPD patients, host defense is impaired by deficiencies of innate immune functions such as phagocytosis and by reduced barrier functions and tissue damage associated with chronic inflammation and repeated pulmonary infections [3]. The inflammatory response after NTHi infection has been characterized by the upregulation of proinflammatory cytokines like IL-1β, CXCL-2 and TNF-α which is mediated by activation of mitogen-activated protein kinases (MAPK) and NFκB through *Toll-like receptor* (TLR) signaling. In addition recent findings suggest the involvement of other *pattern recognition receptors* (PRRs), in particular NOD-like receptors (NLR) in airway inflammation [4], [5].

The IL-1 family of cytokines has a unique, highly preserved way of activation. NLRs can associate with other proteins, forming the inflammasome complex. Inflammasomes are known to control the processing of IL-1β, IL-18 and IL-33 in response to inflammatory stimuli by activation of caspase-1 [6]. Besides their role in the antimicrobial host defense they have been reported to be involved in a wide variety of autoinflammatory diseases such as crystal arthropathies, amyloidosis, periodic fever syndromes and rheumatoid arthritis [7], [8]. However, there has not been much attention to the role of the inflammasome in chronic airway inflammation.

Regarding the impact of pathogen induced AE-COPDs on the course of airway inflammation the aim of our study was to elucidate whether the inflammasome is involved in the host response to respiratory tract infection with NTHi and, if so, which specific set of proteins is upregulated during bacterial stimulation. Our data show enhanced expression of the NLRP3 inflammasome and a strong IL-1 response to viable but not to non-viable microorganisms.

## Methods

### Cell and Tissue Culture

RAW 264.7 cells were cultured at 5% CO_2_ and 37°C in RPMI 1640 medium (PAA Laboratories, Austria) supplemented with 10% not heat-inactivated fetal calf serum (FCS) (PAA/GE Healthcare, Germany), 50 IU/mL penicillin, 100 µg/mL streptomycin and 50 g/L glucose. Cells were spread every 3–4 days and seeded at a number of 1000000 cells/well in 6-well-plates and at a number of 500000 cells/well in 12-well-plates (Cellstar®, greiner bio-one, Germany).

Human lung tissue (HLT) was obtained from ten patients who underwent lung resections for pulmonary nodules as previously described [9]. Specimens were tumor-free material at least 5cm away from the tumor front. Pieces of 0.3–0.4 g were cultured in RPMI 1640 medium (Biochrome AG, Germany) containing 10% FCS in 48-well-plates (nunc, Denmark).

Human lung macrophages were isolated from bronchoalveolar lavage fluids and cultivated as previously described by Davies and Gordon [10].

### Clinical Patient Data

Lung tissue was obtained from ten patients (6 male, 4 female) undergoing lung resection surgery. Mean age was 65±10 years, four patients were active smokers, three were ex-smokers and three were nonsmokers. COPD was confirmed by spirometry according to international guidelines (Global Initiative for Chronic Obstructive Lung Disease, GOLD) in six patients (moderate COPD [formerly GOLD II], n = 5, severe COPD [formerly GOLD III], n = 1; four patients had clinical symptoms of chronic bronchitis, but did not meet the criteria for the diagnosis of COPD. Surgery was only performed in the infection-free interval. None of the patients received systemic glucocorticosteroid treatment.

### Culture of NTHi and Infection Protocol

NTHi (strain Rd KW 20) was taken from fresh overnight culture plates of chocolate-agar and suspended in sterile NaCl solution to a photometric density of 0.5 McF which is equivalent to about 10^8^cfu/ml. This solution was diluted to 10^6^cfu/ml or 10^5^cfu/ml with medium.

Cell and tissue cultures were stimulated for 24–48 h. After 24 h cells were collected for Western Blot analysis and after 24 h and 48 h supernatants were collected for Elisa. For inhibition of caspase-1, cells were incubated with Z-YVAD-FMK 100 µM (Calbiochem, Germany), a specific and irreversible caspase-1 inhibitor, 6 h after being stimulated with NTHi. As a model of the second stimulus for inflammasome activation, nigericin 10 µM (Calbiochem, Germany) was added to NTHi-stimulated lung tissue for 30 min in selected experiments as well as inhibitors of specific pathways (ROS inhibition: RAW cells were pretreated for 1 h with NAC 20 mM before infection with NTHI 10^5^ cfu/ml; blocking of potassium efflux: KCl 30 mM was added 30 minutes prior to stimulation with NTHi 10^6^ cfu/ml; blocking of lysosomal leakage: RAW cells were preincubated for 30 minutes with glibenclamide 250 µM before stimulation with NTHi 10^6^ cfu/ml).

### NTHi Inactivation Protocol

NTHi 10^6^cfu/ml suspensions were prepared as described above. Suspension underwent ten freeze-thaw-cycles being frozen at −80°C and unfrozen at 60°C in a water bath. To test bacterial viability 100 µL from the suspension were plated on chocolate-agar culture plates and incubated for 48 h. Stimulation with inactivated NTHi was only performed if colony growth on culture plates was negative.

### Cytokine Assays

Cell supernatants were collected as described above, pooled and centrifugated to remove cellular debris. Later, supernatants were stored as aliquots at −70°C until use.

Cytokine assays of IL-1β, CXCL-2 and TNF-α were performed with DuoSet® Elisa from R&D Systems as described by the manufacturer. Human IL-18 Elisa Kit was procured from MBL, Japan. Aliquots from at least three experiments were measured in duplicates.

### Western Blot

Cells were collected in lysisbuffer (3,94 g Tris-HCl pH 7,8 (125 mM), 140 ml Aqua dest, 20% Glycerol, 4% SDS, 10% 1 M DTT, brome-phenol-blue; ad 200 ml) and stored at −20°C until use. Later, proteins were separated by SDS-gel electrophoresis, blotted on a Protran® Nitrocellulose Transfer Membrane (Schleicher & Schuell, Germany) and blocked with fat-free milk before incubating with the first antibody for NLRP3 (Adipogen, USA), caspase-1 (Imgenex, San Diego, USA) and β-actin (Cell Signaling, USA) over night. Then, membranes were washed and incubated with the second antibody (HRP-linked anti-rabbit and HRP-linked anti-mouse, Cell Signaling) and measured by the Imager Fusion FX7 (Vilber-Lourmat, Eberhardzell, Germany). NLRP3 protein amount was normalized to the intensity of β-actin bands and was computed by BIO-1D software (Vilber-Lourmat, Germany).

### Real-time Polymerase Chain Reaction (rtPCR) mRNA Expression

RT-PCR was performed using NucleoSpin RNA II kit (Macherey-Nagel, Dueren, Germany) and reverse transcribedinto cDNA (Roche First- Strand PCR kit, Mannheim,Germany), PCR amplification was performed using LightCyclerR Detection System (Roche MolecularBiochemicals, Penzberg, Germany). Oligonucleotide primer specific for mouse NLRP3 (fwd:5′-AATGCTGCTTCGACATCTCC-3′; rev:5′-CCAATGCGAGATCCTGACAA-3′), mouse IL-1β (fwd: 5′-GGTGTGTGACGTTCCCATTA-3′; rev: 5′-GGCCACAGG-TATTTTGTCGT-3′), mouse caspase-1 (fwd: 5′-GTGGAGAGAAACAAGGAGTGG-3′; rev: 5′-AATGAAAAGTGAGCCCCTGAC-3′) and mouse ASC (fwd: 5′-TGAGCAGCTGCAAACGACTA-3′; rev: 5′-CACGAACTGCCTGGTACTGT-3′) were procured from MOLBIOL (Berlin). Cycles of amplification for IL-1β ranged between 18 and 28.

### Immunohistochemistry/Immunocytochemistry

After stimulation, lung tissues and cytospin slides from human lung macrophages were fixed and paraffin-embedded using the HOPE- (*Hepes-Glutamic acid buffer mediated Organic solvent Protection Effect*) technique. Immunohistochemistry was performed as previously described by Droemann et al. [11]. For immunohistochemistry and immunocytochemistry, we used a NLRP3 rabbit monoclonal antibody (Epitomics, Burlingame, USA) and a mouse monoclonal antibody for the detection of caspase-1 protein (Imgenex, San Diego, USA).

### Ethics and Statistical Analysis

Participants of the study were educated orally and in written form about the procedures and informed about the aims of the study. Written consent was obtained. This study was approved by the ethical committee of the University of Luebeck (reference number 08–131) and is in compliance with the Helsinki declaration. Statistic analysis was performed with SPSS 20.0 for Windows using the parametric Student’s t-test and analysis of variance with post-hoc testing for multiple comparisons. Results are shown as mean ± SEM if not stated otherwise. P-values <0.05 were considered as statistically significant.

## Results

**Figure 1:**
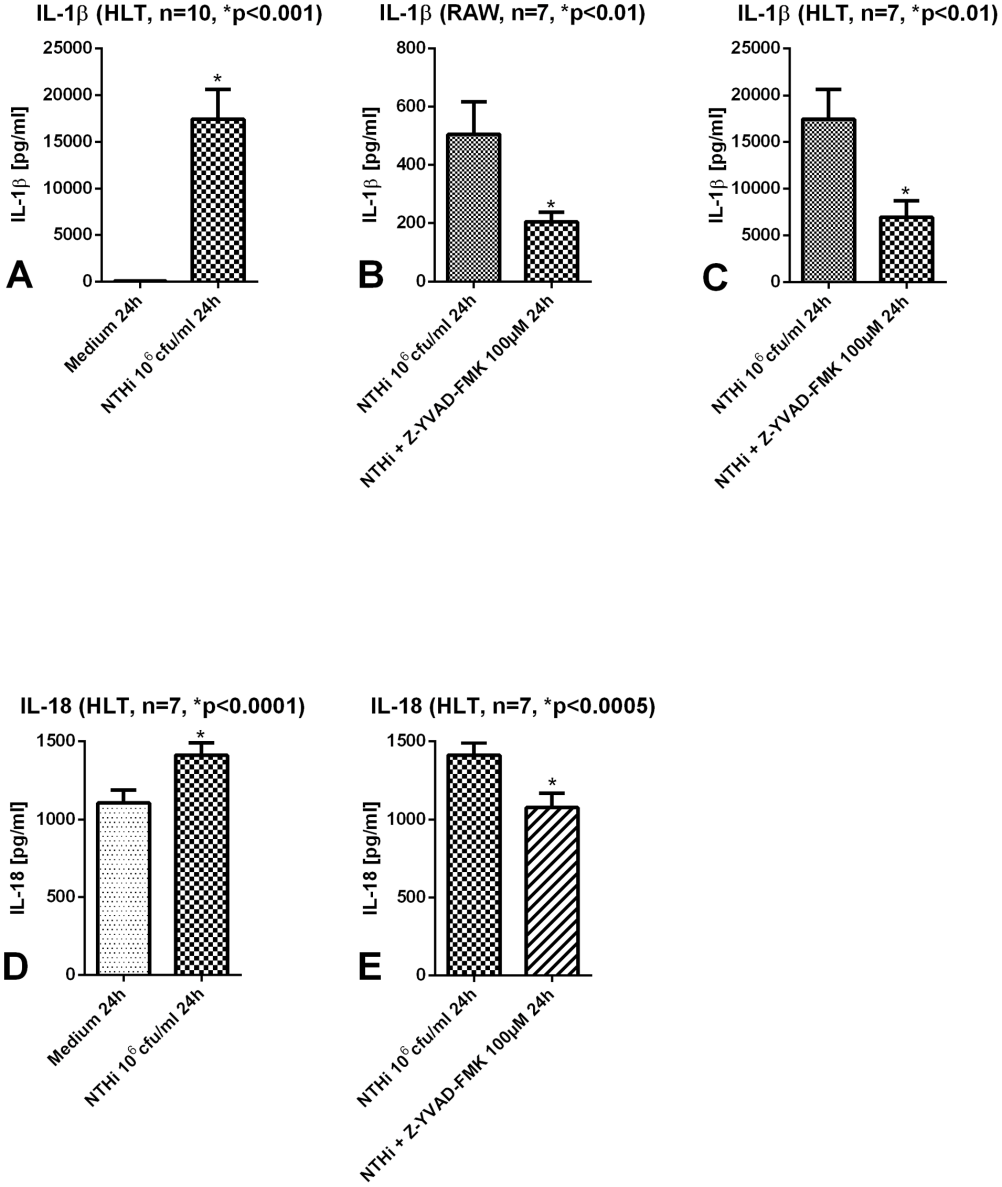
Cytokine secretion in macrophages and human lung tissue after stimulation with NTHi. Human lung tissue (HLT) and murine macrophages were stimulated for 24 h with NTHi 10^6^ cfu/ml. IL-1β and IL-18 concentrations were significantly increased in HLT (A+D). 6 h after stimulation with NTHi a caspase-1 inhibitor (Z-YVAD-FMK 100 µM) was added. After inhibition IL-1β and IL-18 concentrations in HLT and macrophages were significantly reduced (B,C and E).

Figure 1A-E show concentrations of IL-1 family cytokines IL-1β and IL-18 in supernatants of murine macrophages and human lung tissue after stimulation with NTHi with and without inhibition of caspase-1. As expected, NTHi infection led to a marked IL-1β release. To elucidate the effect of caspase-1 on IL-1β production, cells and lung tissue were incubated with the caspase-1 inhibitor Z-YVAD-FMK 6 h after NTHi infection. We could show that caspase-1 inhibition leads to a significant reduction of IL-1β secretion in RAW cells and in human lung tissue (Fig. 1B+C). IL-18 which is constitutively expressed [12] showed comparatively higher basal amounts in tissue supernatants but also a significant, caspase-1 dependent increase after NTHi stimulation (Fig. 1D+E).

### IL-1 Response Requires Viable Bacteria

**Figure 2:**
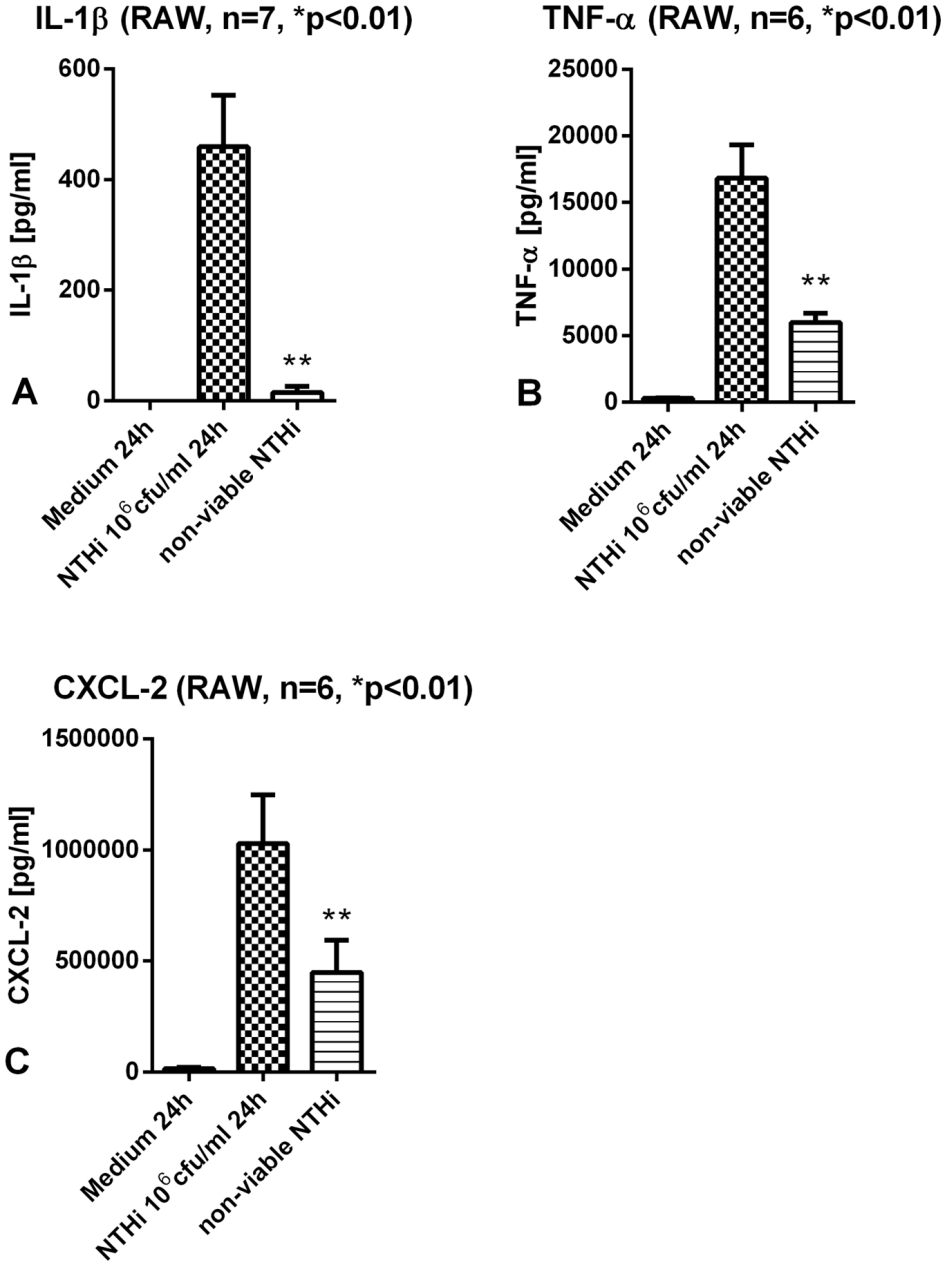
Cytokine secretion in murine macrophages after stimulation with non-viable NTHi. Murine macrophages were stimulated with inactivated NTHi for 24 h. IL-1β concentrations were significantly reduced compared to stimulation with viable NTHi (A). TNF-α and CXCL-2 levels were better preserved after stimulation with non-viable NTHi in comparison to IL-1β (B+C).

The secretion of IL-1β after stimulation with non-viable NTHi was significantly reduced tending to no response at all in comparison to infection of RAW with viable NTHi. Other inflammatory cytokines like TNF-α and CXCL-2 showed as well a significant decrease of concentrations but were better preserved than IL-1β after challenge with non-viable NTHi (Fig. 2A-C). mRNA levels of IL-1β are elevated after stimulation with non-viable NTHi to a lesser degree than after viable NTHi (Fig. S1). However the bio-active form of IL-1β protein was not secreted by the macrophages.

### NTHi Induces Protein Components of the NLRP3 Inflammasome

**Figure 3:**
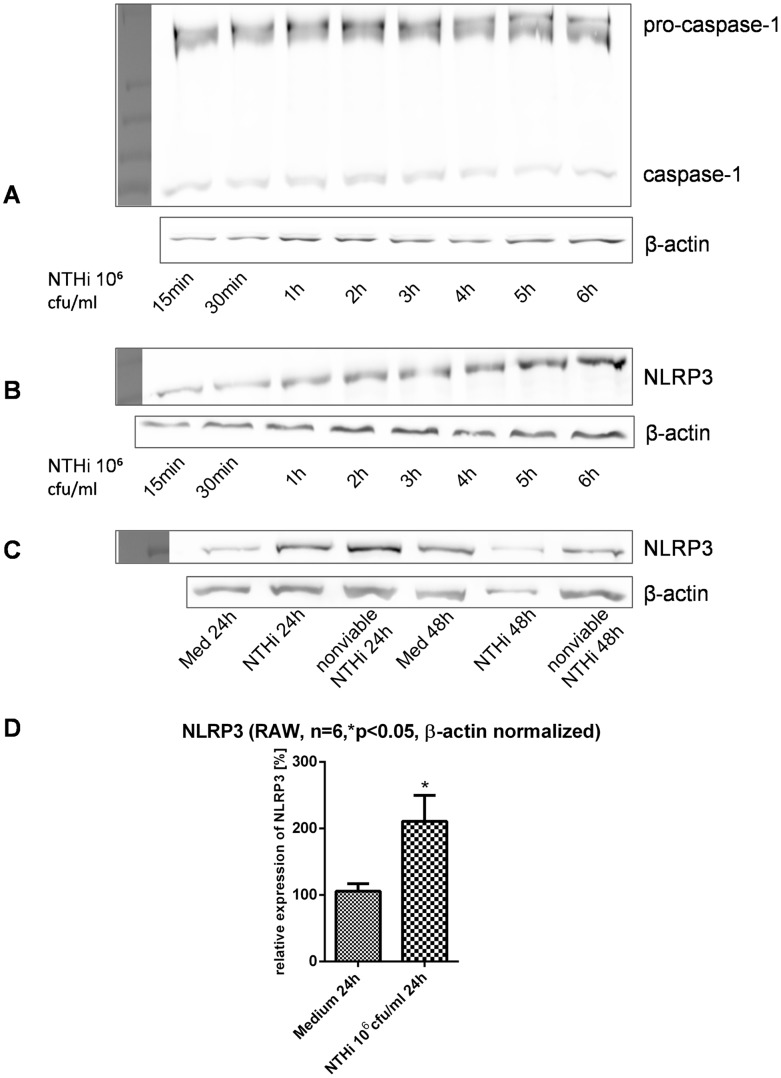
Western Blot analysis of inflammasome components after stimulation with NTHi. A+B show caspase-1 (A) and NLRP3 (B) expression over a period of 6 h after stimulation with NTHi 10^6^ cfu/ml in murine macrophages. NLRP3 was detected in murine macrophages after stimulation with inactivated NTHi as well (C). Densitometric analysis of NLRP3 in murine macrophages after stimulation with NTHi 10^6^ cfu/ml for 24 h is shown in (D).

Next we elucidated which specific set of inflammasome components is upregulated after stimulation with NTHi. NLRP3 inflammasome key proteins NOD-like receptor NLRP3, and caspase-1 were detected via Western Blot in murine macrophages. NLRP3 was significantly upregulated after NTHi treatment (Fig. 3D).

**Figure 4:**
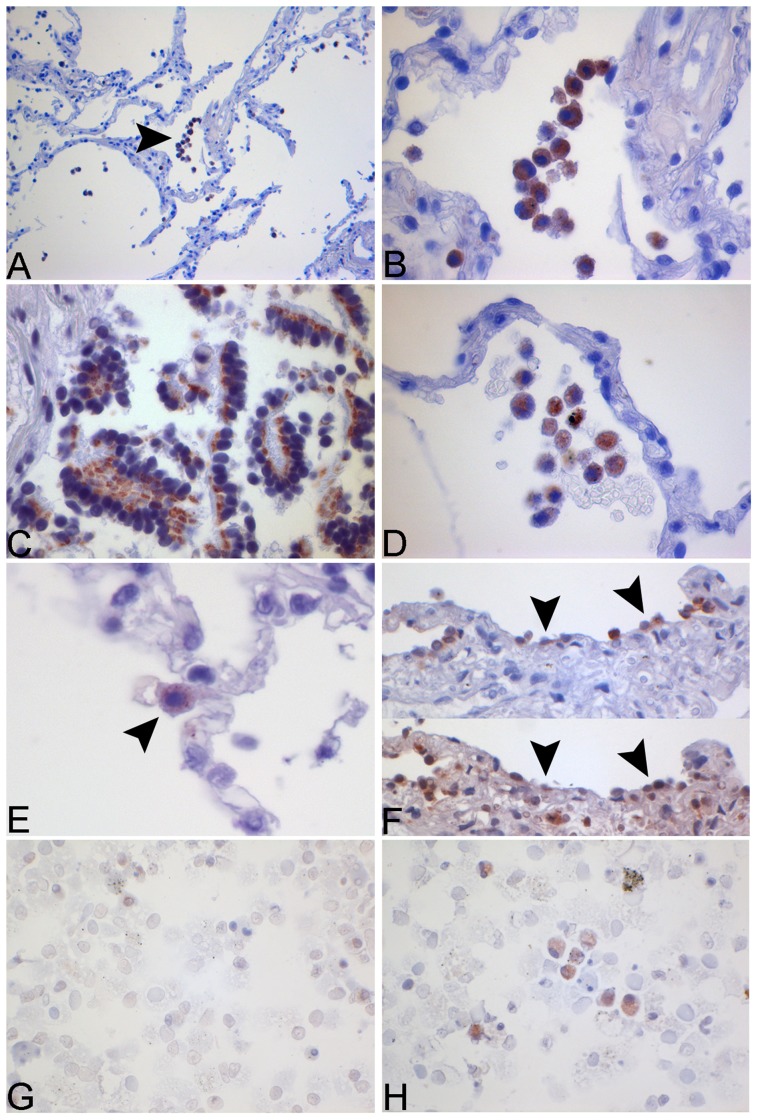
Immunohistochemical and immunocytochemical staining for NLRP3 in human lung tissue and human alveolar macrophages. Human lung tissue was stimulated with NTHi 10^6^ cfu/ml for 24 h. Tissue was fixated with HOPE-solution and NLRP3 detected via IHC. The images show the expression of NLRP3 in alveolar macrophages (A+B), bronchial epithelial cells (C), in the medium control (D) and in alveolar type II cells (ATII) (E). A specific ATII staining (human surfactant protein B) was performed in HLT (F, upper image). NLRP3-positive cells (F, lower image) were identified in consecutive slides from the same tissue section (arrows). Human alveolar macrophages from BAL were stained for NLRP3. Increased expression can be observed after stimulation with NTHi (H) in contrast to the medium control (G).

**Figure 5:**
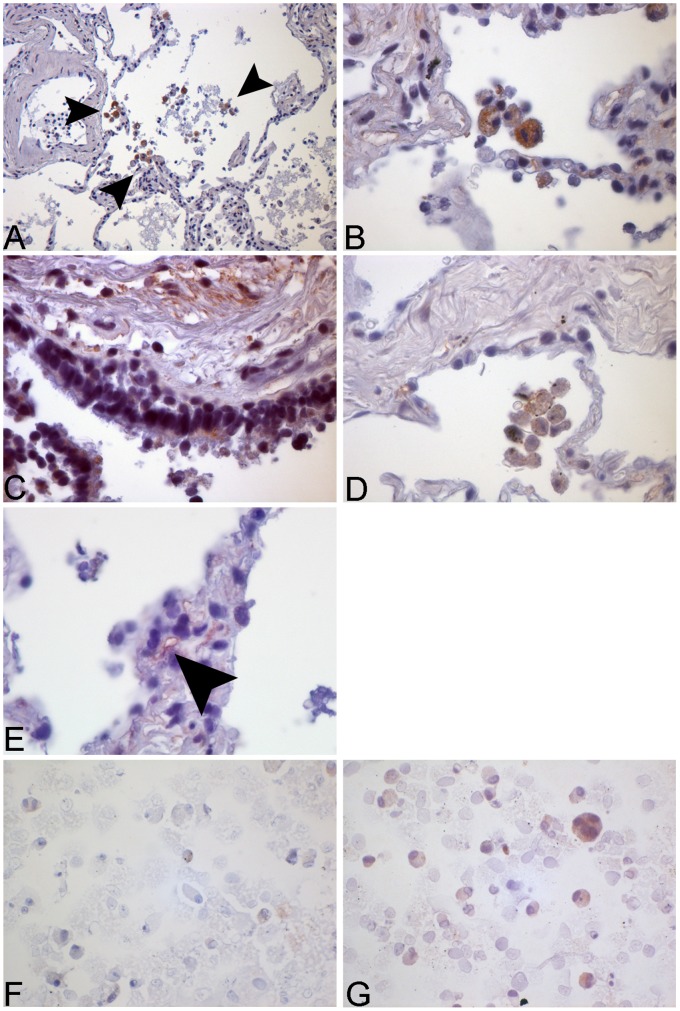
Immunohistochemical and immunocytochemical staining for caspase-1 in human lung tissue and human alveolar macrophages. Human lung tissue was stimulated with NTHi 10^6^ cfu/ml for 24 h. Tissue was fixated with HOPE-solution and caspase-1 detected via IHC. The images show the expression of caspase-1 in alveolar macrophages (A+B), bronchial epithelial cells (C), in the medium control (D) and in the capillary endothelium (arrow) (E). Human alveolar macrophages from BAL were stained for caspase-1. Increased expression can be observed after stimulation with NTHi (G) in contrast to the medium control (F).

NLRP3 and caspase-1 were not only detected in murine macrophages, but for the first time we could show the localization of inflammasome components in human lung tissue (Fig. 4 and 5).The upstream NOD-like-receptor NLRP3 was present in alveolar macrophages in the unstimulated control group tissue (Fig. 4D) indicating a constitutive expression of this protein in unchallenged airways. After stimulation with NTHi staining within the alveolar macrophages intensified. The same expression pattern and stimulation after NTHi infection was shown in primary human macrophages derived from bronchoalveolar lavage (Fig. 4G+H).

Interestingly, in human lung tissue not only macrophages in the alveolar air space were positive to staining, but expression of NLRP3 was also seen in bronchial epithelial cells and alveolar type II cells (ATII) showing intensification after NTHi infection (Fig. 4C, E and F).

As described above for NLRP3 also the protein caspase-1 was found to be expressed in alveolar macrophages as well as in bronchial epithelial cells with increased staining after stimulation (Fig. 5A-D, F+G). In addition we found caspase-1 expression in the alveolar capillary endothelium in stimulated lung specimen (Fig. 5E).

### DAMPs are a Second Hit for Inflammasome Activation

**Figure 6:**
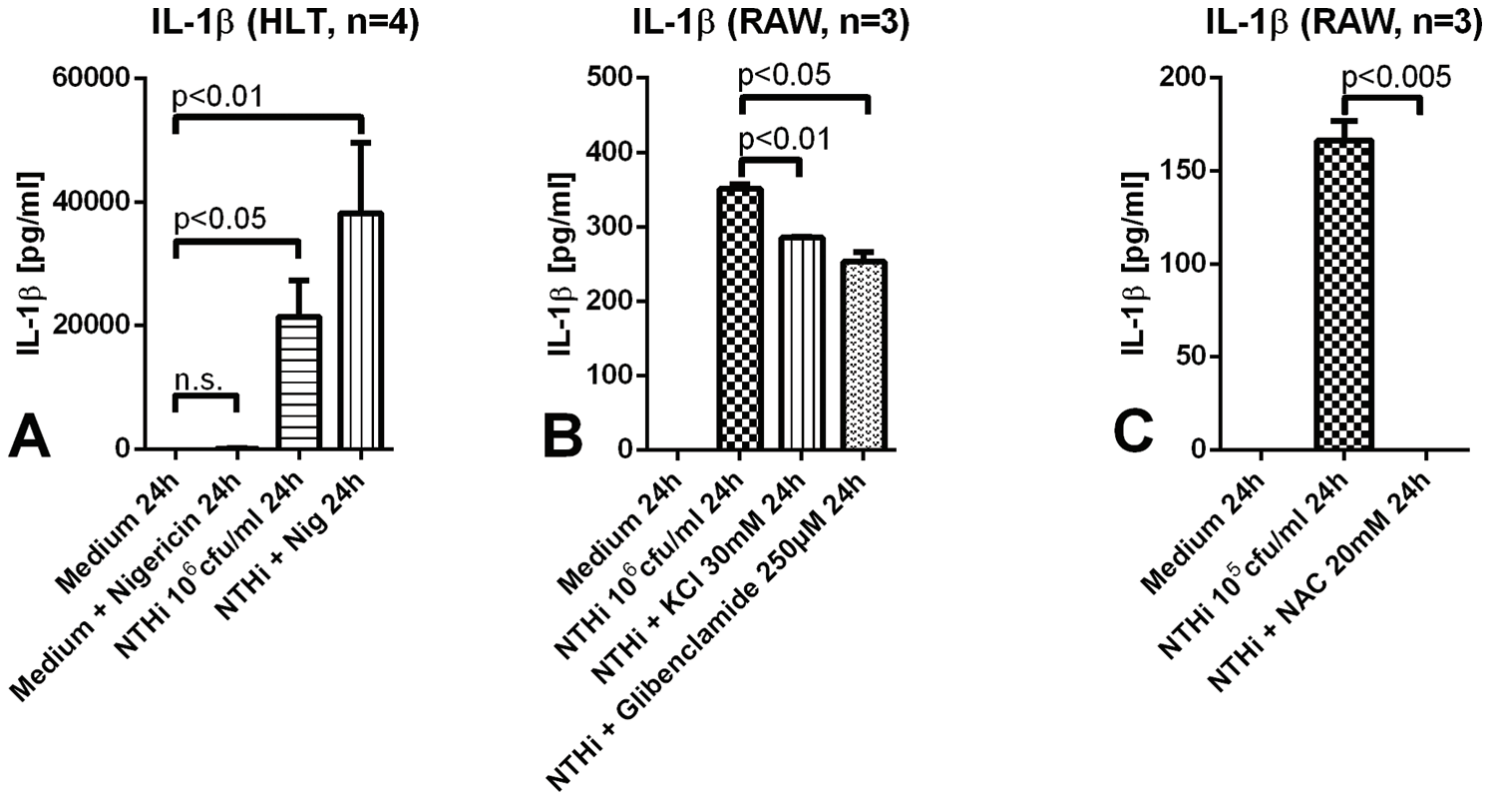
Cytokine secretion in human lung tissue and murine macrophages after challenge with nigericin, KCl, glibenclamide and NAC. A. Human lung tissue was stimulated either with nigericin 10 µM alone or together with NTHi 10^6^ cfu/ml. Sole challenge with nigericin did not lead to elevated IL-1β levels, whereas the addition of nigericin to NTHi primed macrophages enhanced the cytokine secretion; (n.s. = not significant). B. RAW cells were pretreated for 30 minutes with KCl 30 mM or glibenclamide 250 µM and then stimulated with NTHi 10^6 ^cfu/ml. IL-1β concentrations decreased significantly in both settings. C. RAW cells were preincubated with NAC 20 mM for 60minutes prior to stimulation with NTHi 10^5^ cfu/ml. IL-1β levels were reduced significantly in comparison to the control.

To test the effect of cell damage associated stimuli as a second hit for inflammasome activation, we used nigericin (Streptomyces hygroscopicus), a potassium ionophore, to establish a model for this clinically relevant situation. Although we performed only a limited number of experiments wherein human lung tissue was stimulated either with nigericin alone or together with NTHi, a clear tendency can be observed. Our data indicate that a primary microbial challenge is needed for a robust IL-1 response although a second stimulus can enhance its release (Fig. 6A). A role for reactive oxygen species, potassium efflux and lysosomal leakage is suggested by significant effects of inhibitors of these mediators and pathways on IL-1 release (Fig. 6B, C).

## Discussion

**Figure 7:**
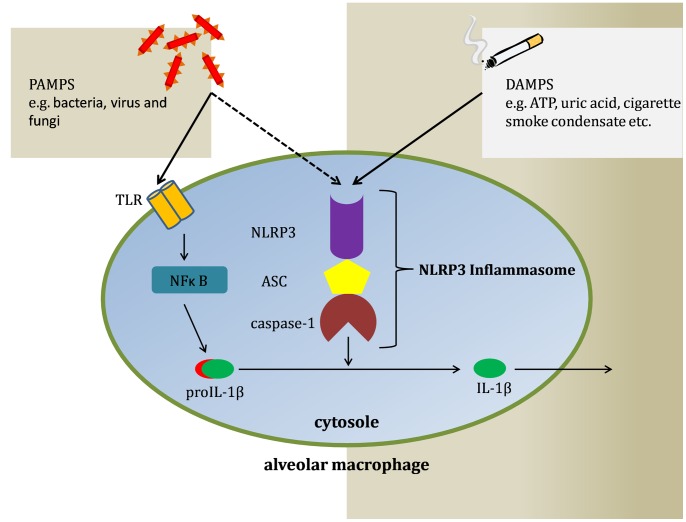
Overview of inflammasome activation. Two different types of stimuli are able to activate the inflammasome, pathogen associated molecular patterns (PAMPs) on the one hand and damage associated molecular patterns (DAMPs) on the other. A 2-hit-theory has been postulated, stating that for inflammasome activation two distinct signals are required. Only thereafter proIL-1β is cleaved by caspase-1, so that mature IL-1β can be secreted by macrophages and promote the inflammatory response.

Inflammasomes are a heterogenous group of proteins recruited to promote inflammation. Dependent from the tissue and stimulus, different types of inflammasomes are activated [13]. The NLRP3 inflammasome consists of the NOD-like receptor NLRP3, the adaptor molecule ASC (*apoptosis-associated speck-like protein containing a CARD, caspase-associated recruitment domain*), and the cysteine protease caspase-1, which is responsible for the maturation of IL-1β, IL-18 and IL-33. The NLRP3 inflammasome can be activated by different stimuli, including respiratory viruses, gram-positive and gram-negative bacteria and fungi [14]–[16]. We could demonstrate for the first time that the NLRP3 inflammasome is upregulated by NTHi. Moreover the data from our human lung tissue infection model show that NTHi stimulates caspase-1 expression and leads to a strong release of IL-1 family cytokines IL-1β and IL-18. Thus different signal pathways appear to be induced by the same pathogen (Fig. 7). Several microbial signals are recognized by TLRs and intracellular *nucleotide-binding oligomerization domain* (NOD1, NOD2) receptors leading to activation of the MAPK-pathway and NFκB [17], [18]. These transcription factors enhance the expression and synthesis of proIL-1β, but do not drive the activation of the inflammasome. Consequently, another NLR family must be responsible for the assembly of the inflammasome and maturation of IL-1β. In case of stimulation with NTHi NLRP3 may recognize PAMPs such as muramyl dipeptide amongst other, or danger signals like mRNA, uric acid, toxins, and ATP. However, the transcriptional upregulation of NLRP3 detected in this study does not prove that the inflammasome is activated.

Pneumolysin, an important virulence factor of *Streptococcus pneumoniae*, has been proven to activate the NLRP3 inflammasome [19]. Panton-Valentine-Leucocidin, a virulence factor implicated in necrotic staphylococcal pneumonia, induces inflammasome upregulation and IL-1β release in human alveolar macrophages [20]. In an experimental model of *Klebsiella pneumoniae* pulmonary infection the NLRC4 inflammasome was shown to be important for host defense and survival [21]. He and coworkers showed that TLR2 activation was required for proIL-1β production in response to stimulation with *Chlamydia pneumoniae*, whereas NLRP3 inflammasome activation was required for caspase-1 activation and processing of proIL-1β into its active form IL-1β [22]. Thus, a first signal activating TLR or other receptors is necessary to induce transcription of proinflammatory cytokines, whereas the second stimulus induces inflammasome activation and posttranslational processing of IL-1β [23]. Recently, TIR-domain-containing adapter-inducing interferon-β (TRIF) has been discussed as a possible link between the TLR pathway and the NLRP3 inflammasome [24].

To evaluate the role of the NLRP3 inflammasome in chronic airway diseases it is important to characterize the expression sites of the inflammasome components. Research has been mostly focused on macrophages. In this study we not only identified inflammasome components NLRP3 and caspase-1 in murine and human alveolar macrophages, but also in human bronchial and alveolar epithelial cells (AEC). The presence of inflammasome components in AECs expands on prior findings concerning the role of the epithelium in innate immunity and host defense [25]. The auxiliary finding of inflammasome components in alveolar capillary endothelium leaves room for speculations about the contribution of vascular endothelium in alveolar tissue inflammation. These findings are supported by work done by Tran et al. detecting NLRP3 inflammasome components in healthy and inflamed murine airway epithelium [26]. In contrast, NLRP3 mRNA could not be detected in human respiratory epithelia derived from bronchial brushing [30]. Cohen et al. depleted murine BAL-macrophages which resulted in a significant decrease of IL-1β and IL-18 levels [27]. Nevertheless, both cytokines were still preserved indicating a macrophage-independent IL-1β secretion.

Since the respiratory tract is permanently exposed to inhaled antigens, a crucial question is how the potency of inflammatory responses is adjusted. We here show that discrimination between viable and non-viable microbial stimuli plays a decisive role. Although stimulation with non-viable NTHi led to the release of TNF-α and CXCL-2 and induced the expression of NLRP3, supernatants showed marginal evidence of IL-1β release although IL-1β mRNA was present regardless of the viability of the microorganism. These findings suggest that the inflammasome had not been activated. Our results support findings reviewed recently by Blander and Sander that only viable bacteria, expressing so-called ‘vita PAMPs’ promote inflammasome activation; phagocytosis of bacteria and the subsequent release of mRNA are crucial in this respect [28]. This is in accordance with data from cell culture models and in knock-out mice that live bacteria are required for activation of the NLRP3 inflammasome and IL-1β-processing [29].

### Danger Signals for Inflammasome Activation

IL-1β is one of the most potent proinflammatory cytokines. Apart from pulmonary infections noninfectious injuries like mechanical ventilation are able to induce expression of the NLRP3 inflammasome; inflammasome dependent lung injury is reduced in NLRP3 deficient mice [30]. Thus, the expression and secretion of IL-1β need to be tightly regulated and it is important to characterize the checkpoints where this regulation takes place. A two-hit theory has been postulated, proposing a microbial stimulus as a ligand for recognition receptors and a second stimulus (*damage-associated molecular pattern*, DAMP) like ATP, potassium efflux, lysosomal leakage or production of reactive oxygen species (ROS) as trigger for the inflammasome complex in cells previously primed by the microbial antigen [31]–[33]. Our data show that the IL-1β response is amplified by a challenge with nigericin, a potassium ionophore. Correspondingly, inhibition of potassium efflux reduced IL-1 release significantly. However, a mere potassium efflux appears to be a subordinate signal and a microbial hit is needed for the activation since nigericin alone was not able to elicit a significant IL-1 response. In line with previous investigators IL-1 release could be completely blocked by inhibition of ROS with N-acetyl-cysteine. ROS inhibition was only successful when performed before NTHi stimulation (data not shown), suggesting a predominant effect of ROS inhibition on priming by proinflammatory signals [12].

COPD is characterized by chronic inflammation and tissue damage including apoptosis of alveolar cells. Thus the microenvironment in the lower airways is enriched with danger signals and DAMPs may be considered as a relevant pathogenetic factor of the disease [34]. In this context cigarette smoke plays an important role [35]. Cigarette smoke-induced tissue damage and acidification favor the formation of uric acid and calcium pyrophosphate, both activators of the NLRP3 inflammasome [36]. Moreover, extracellular ATP which is increased in chronic inflammation binds to the purinergic receptor P_2_X_7_ acting as a DAMP as well [37]. Doz et al. showed that cigarette smoke condensate itself promoted an inflammasome driven inflammatory response [38]. An open question is whether a second microorganism can replace the cellular DAMP leading to enhanced stimulation of the inflammasome. This may be particularly relevant in viral-bacterial coinfections which are frequent in COPD exacerbations and are associated with a more severe course of disease [39].

### Conclusion

Upregulation of the NLRP3 inflammasome by NTHi together with a strong IL-1β driven inflammatory response suggest an involvement of this pathway in the pathobiology of COPD. This may lead to reanalysis of the role of antiinflammatory treatment options in chronic airway disease. The limited effectiveness of glucocorticosteroids (GC) in the management of COPD may be explained by the fact that GC have an effect on cytokine transcription but not posttranslational steps like IL-1β maturation [40]. Ultimately, the regulation of the inflammasome offers interesting new pharmaceutical targets for immunoregulatory therapies. These comprise the upstream antagonization of purinergic receptor P_2_X_7_ as well as the IL-1 receptor [36], [37]. As other authors have suggested the reduction of IL-1β concentrations as a therapeutic target, the inhibition of caspase-1 may also come into consideration [41].

## Supporting Information

Figure S1 **PCR data of IL-1β mRNA.** Murine macrophages (RAW 264.7) were stimulated with NTHi 10^6^ cfu/ml (A) or with nonviable NTHi (B) for 15 minutes to 6 hours.
(TIF)
