# Supplementary figures and images for: Nontypeable Haemophilus Influenzae Infection Upregulates the NLRP3 Inflammasome and Leads to Caspase-1-Dependent Secretion of Interleukin-1β — A Possible Pathway of Exacerbations in COPD
Source: PLoS One. 2013 Jun 26;8(6):e66818. doi: 10.1371/journal.pone.0066818 (PMC3694113; doi:10.1371/journal.pone.0066818)

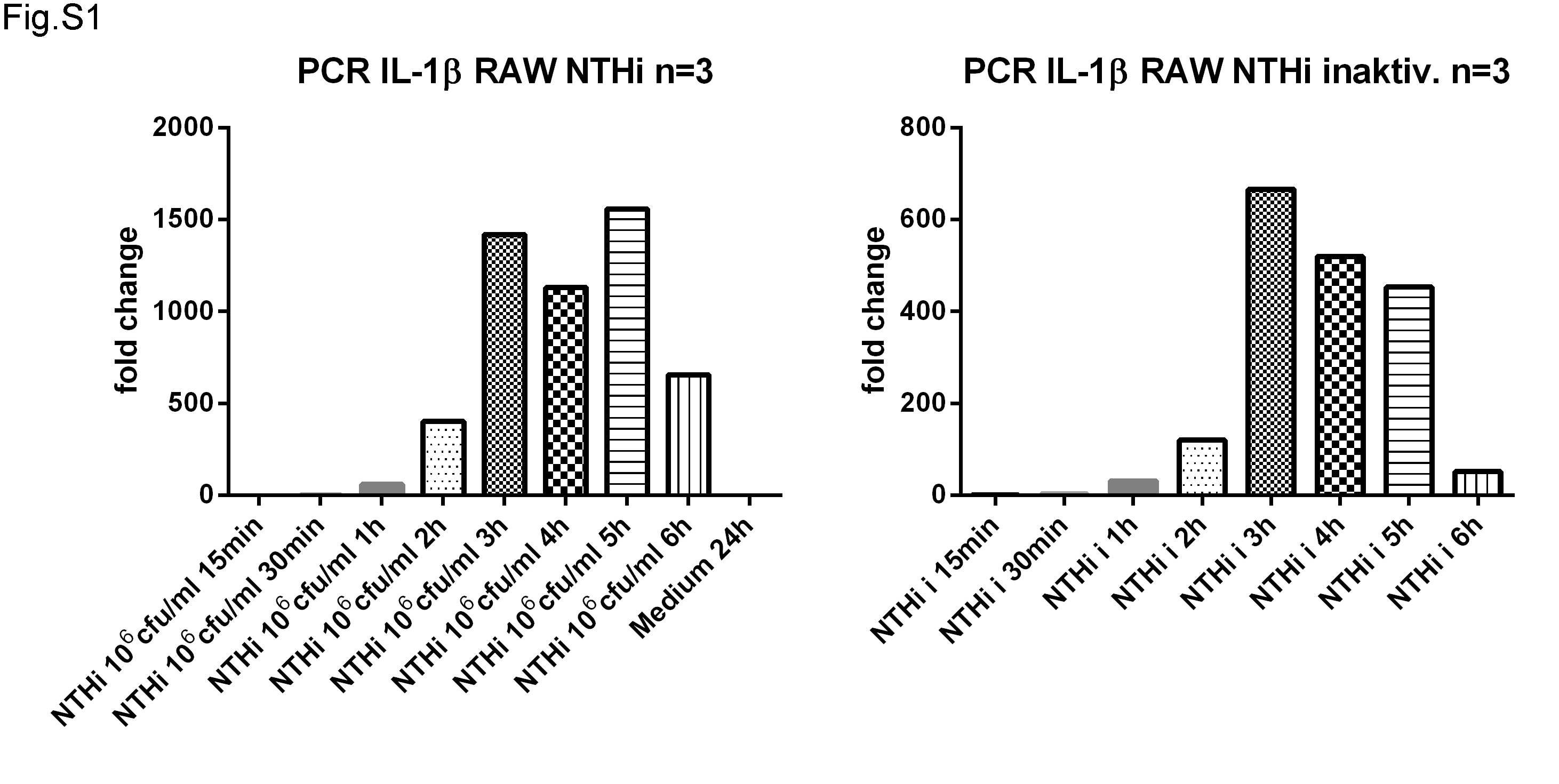

Supplement: Figure S1 — PCR data of IL-1β mRNA. Murine macrophages (RAW 264.7) were stimulated with NTHi 106 cfu/ml (A) or with nonviable NTHi (B) for 15 minutes to 6 hours. (TIF) [file pone.0066818.s001.tif]
